# Interleukin-17 and interleukin-10 in the adenoid hypertrophy children concomitant with otitis media with effusion

**DOI:** 10.3389/fphar.2026.1743884

**Published:** 2026-04-07

**Authors:** Yuyan Yan, Lujie Zuo, Yafang Wang, Li Cui, Yingluan Song, Li Wang, Xiaofeng Liu, Hongbo Wang, Jiangqiao Geng

**Affiliations:** Department of Otolaryngology, Head and Neck Surgery, Children’s Hospital of Hebei Province, Hebei Provincial Clinical Research Center for Child Health and Disease, Shijiazhuang, Hebei, China

**Keywords:** adenoidal hypertrophy, cytokines, interleukin-10, interleukin-17, otitis media with effusion

## Abstract

**Purpose:**

Adenoid hypertrophy (AH) is a prevalent cause of otitis media with effusion (OME) in children, although its pathogenesis remains poorly understood. The objective of this study is to assess the levels of interleukin-17 (IL-17) and interleukin-10 (IL-10) in children with isolated AH and those with AH in conjunction with OME.

**Methods:**

Thirty children with AH and OME who underwent adenoidectomy and tympanostomy tube placement were included in the OME group, whereas 30 children with isolated AH who underwent adenoidectomy were included in the AH group. Adenoid tissue samples were collected during surgery, and preoperative venous blood samples were acquired. Concentrations of IL-17 and IL-10 in both adenoid tissue and serum were measured using enzyme-linked immunosorbent assay (ELISA).

**Results:**

Levels of IL-17 (4.20 ± 1.51 ng/mL) and IL-10 (3.77 ± 1.06 ng/mL) in adenoid tissue were significantly lower in the OME group than in the AH group (5.29 ± 2.16 ng/mL and 4.80 ± 1.93 ng/mL, respectively; P < 0.05). No significant differences in serum levels of IL-17 or IL-10 were observed between the two groups (P > 0.05). ROC analysis revealed that adenoid tissue IL-17 and IL-10 exhibited higher AUC values, specificity, and sensitivity for predicting AH in combination with OME compared to their serum counterparts (P < 0.05).

**Conclusion:**

The reduced local expression of IL-17 and IL-10 in adenoid tissue is associated with the development of OME in children with AH. Targeting these cytokines may provide potential therapeutic strategies for preventing or treating AH-related OME.

## 1 Introduction

Otitis media with effusion (OME), also known as secretory otitis, is a prevalent childhood disease that has become increasingly common over the last decade. This condition may lead to severe, irreversible changes in the middle ear, resulting in conductive hearing loss and subsequent impairment of speech and language development (Zielnik-Jurkiewicz and Stankiewicz-Szymczak, 2016; Qureishi et al., 2014). It often presents a significant source of distress and financial burden for children and their families. Identifying the underlying cause of OME is crucial for its treatment.

It is well established that adenoid hypertrophy (AH) is a significant pathogenic factor in the development of OME in children. Clinically, severe complications of OME have been observed in children with AH. Adenoid resection remains a crucial treatment for refractory otitis media. However, clinical findings indicate that there is no direct positive correlation between the degree of AH and the presence of OME. The factors influencing AH’s impact on the incidence of OME in children warrant further investigation. Adenoid tissue is part of the lymphoid tissues associated with the upper airway mucosa (MALT), where T cells play a central role in the local immune response (Sade et al., 2011). The middle ear is anatomically adjacent to the adenoids. The mucosal epithelium of the middle ear contains a substantial amount of MALT. The development of mucosa-specific immunity is closely associated with MALT. Thus, the onset of OME complicated by AH may also be associated with the autoimmunity of adenoids, in addition to Eustachian tube obstruction caused by AH. Several studies have focused on OME caused by AH (Zelazowska-Rutkowska et al., 2020; Huang et al., 2019; Żelazowska-Rutkowska et al., 2012). Increasing evidence suggests that adenoid immune dysfunction may induce and even exacerbate OME, although the exact mechanism remains poorly defined (Ni et al., 2015).

The role of helper T cells (Th17) and regulatory T cells (Treg) in maintaining immune homeostasis in adenoid hypertrophy has recently garnered increasing research attention. A study found (Ni et al., 2015) that an altered Th17/Treg cell ratio in local adenoid tissue and peripheral blood of children with AH is associated with disease development. Studies also suggest that Th17 and Treg cells, along with their associated cytokines, play a crucial role in the response to pneumococcal carriage in the nasopharyngeal adenoids (Zhang et al., 2011; Jochems et al., 2017). For many mucosal pathogens, pathogenesis begins with microbial adhesion to the mucosa, followed by transient colonization (Bogaert et al., 2004; Siegel and Weiser, 2015). Each episode of colonization may last from days to months, providing an opportunity for infection. Consequently, the host’s ability to eliminate bacteria from the mucosal surface of the nasopharynx is crucial. Efficient mucosal clearance can reduce the duration of colonization and may even lower the risk of infection. The balance of the Th17 and Treg-mediated immune response regulates pneumococcal carriage in the nasopharyngeal adenoids. However, the role of Th17/Treg immune balance in the pathogenesis of pediatric AH combined with OME remains underexplored.

IL-17 is the primary effector molecule of Th17 cells, promoting T cell activation and stimulating epithelial cells, endothelial cells, and fibroblasts to produce various cytokines, leading to inflammation. It has been reported (Huang et al., 2019) that IL-17A deficiency can lead to the persistent colonization of *Streptococcus pneumoniae* (*S. pneumonia*) in adenoids, resulting in otitis media or promoting its development. IL-10 is an immunosuppressive cytokine that plays a critical role in the immunosuppressive functions of Treg cells, inhibiting T cell proliferation, suppressing inflammatory and immune responses, and modulating immune cell function. The objective of this study was to assess the levels of IL-17 and IL-10 in the adenoids and serum of children with AH and OME, and to explore the significance of these cytokines in children with AH combined with OME.

## 2 Materials and methods

### 2.1 Patients

This prospective study enrolled 30 pediatric patients with OME and AH (OME group) and 30 controls with isolated AH (AH group) at the Children’s Hospital of Hebei Province from March 2023 to April 2024. The sample size was determined based on a power analysis using G*Power software (version 3.1). Based on a pilot study with 10 patients per group, which showed a mean difference in adenoid tissue IL-17 of 1.0 ng/mL with a standard deviation of 1.2 ng/mL, we calculated that a sample size of 24 patients per group would provide 80% power at a two-sided alpha level of 0.05. To account for potential dropouts or sample exclusions, we enrolled 30 patients per group. The age range was from 2 to 10 years. All patients required adenoidectomy, and all OME patients required tympanotomy or tympanostomy tube insertion. Patients were scheduled for adenoidectomy based on a history of nasal obstructive symptoms and fiber nasopharyngoscope findings showing more than 2/3 choanal occlusion, or refractory otitis media with nasal fibroscopy revealing more than 1/2 choanal occlusion. Patients scheduled for tympanostomy tube insertion had unilateral or bilateral OME lasting at least 3 months, accompanied by hearing impairment.

#### 2.1.1 Exclusion criteria

Patients with cardiovascular, endocrine, urinary, metabolic, or neuromuscular diseases were excluded from the study. Additionally, all children had no aspirin hypersensitivity, bronchiectasis, autoimmune diseases, or a history of upper respiratory tract infection or antacid use within 2 weeks.

**FIGURE 1:**
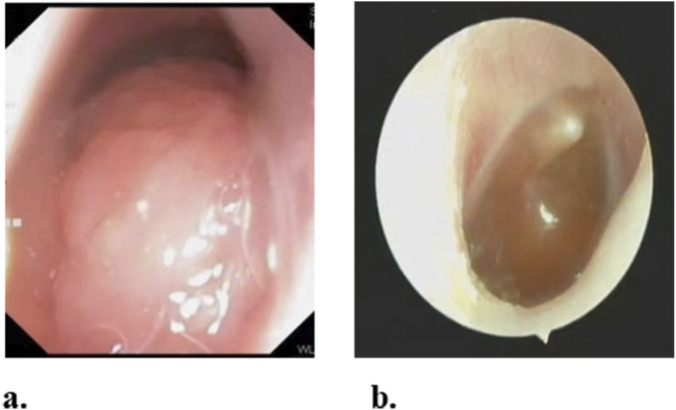
**(a)** By fiber nasopharyngoscope, hypertrophic adenoid was found block choana more than 1/2. **(b)** By ear endoscope, Obvious effusion was found in the tympanic cavity.

The size of the nasopharyngeal adenoids was measured using a fiber nasopharyngoscope (Figure 1a). The OME condition was evaluated using an ear endoscope (Figure 1b). The diagnosis of OME was confirmed by a combination of otoscopy, tympanometry, and audiometry. Tympanometry was performed using a GSI TympStar Pro middle ear analyzer. A flat tympanogram (Type B) or a highly negative pressure tympanogram (Type C with peak pressure < −200 daPa) was considered indicative of middle ear effusion. Audiological evaluation was conducted using pure-tone audiometry (for children aged ≥4 years) or auditory brainstem response (ABR) testing (for children aged <4 years). Hearing impairment was defined as an average pure-tone threshold >20 dB HL over 0.5, 1, 2, and 4 kHz, or an ABR threshold >30 dB nHL. All patients underwent both fiber nasopharyngoscopy and ear endoscopy. Two experienced otolaryngologists participated in diagnosing AH and OME.

### 2.2 Sampling methods

#### 2.2.1 Collection of adenoid specimens

All children underwent low-temperature plasma resection of the adenoids under general anesthesia. Adenoid tissue specimens were obtained from the OME and AH groups during surgery at the Children’s Hospital of Hebei Province. After completion of adenoidectomy, adenoid samples were carefully cleaned with sterile saline and stored at −80 °C for later use.

#### 2.2.2 Collection of serum

2 mL of venous blood was drawn from the OME and AH groups after overnight fasting. The plasma supernatant was obtained after centrifugation for 7 min at 4,000 rpm, and stored at −80 °C until use.

### 2.3 IL-17 and IL-10 concentrations detection in adenoid tissue and serum by enzyme-linked immunosorbent assay (ELISA)

Adenoid tissue processing: Adenoid tissue samples were thawed, weighed, and homogenized in ice-cold phosphate-buffered saline (PBS) at a ratio of 100 mg tissue per 1 mL PBS using a hand-held homogenizer. The homogenates were then centrifuged at 12,000 × g for 20 min at 4 °C, and the supernatants were collected for cytokine analysis. All results were normalized to the total protein concentration of each sample, which was determined using the Bicinchoninic Acid (BCA) Protein Assay Kit (Beyotime Biotechnology, Shanghai, China), to account for variations in tissue sample size and ensure comparability across specimens.

Commercially available ELISA kits were used to detect IL-17 and IL-10 concentrations (Jiangsu Jingmei Biotechnology Company Limited, Jiangsu, China). According to the manufacturer’s specifications, the minimum detectable concentrations were <0.1 ng/mL for IL-17 and <0.05 ng/mL for IL-10. The detection ranges were 0.2–20 ng/mL for IL-17 and 0.1–15 ng/mL for IL-10. The intra-assay coefficients of variation (CVs) were <5% and the inter-assay CVs were <8% for both cytokines, indicating good assay precision and reproducibility.

The specific experimental procedures were performed according to the manufacturer’s instructions. All samples, including standards and blanks, were run in duplicate to ensure accuracy. Absorbance values were measured at 450 nm using a microplate reader (Shanghai Shanpu Biotechnology Co., Ltd., Shanghai, China). Results were classified as positive if IL-17 and IL-10 concentrations were successfully quantified within the linear range of the standard curve.

### 2.4 Statistical analysis

Data analysis and all statistical tests were performed using IBM SPSS Statistics 23. Continuous data are expressed as mean ± standard deviation. Age differences between the two groups, as well as IL-17 and IL-10 concentrations in adenoid tissue and serum, were statistically analyzed using the Student’s t-test. The Chi-square test was used to analyze gender differences and adenoid size differences between the OME and AH groups. To assess whether the observed differences in cytokine levels were independent of potential confounding factors, we performed multivariate binary logistic regression analysis. The dependent variable was group assignment (OME vs. AH), and the independent variables included adenoid tissue IL-17, adenoid tissue IL-10, age, gender, and adenoid size. Adjusted odds ratios (ORs) with 95% confidence intervals (CIs) were calculated. The predictive value of adenoid tissue IL-17, serum IL-17, adenoid tissue IL-10, and serum IL-10 for children with AH combined with OME was analyzed using ROC curve analysis, with 95% confidence intervals calculated for AUC, sensitivity, and specificity.

## 3 Results

### 3.1 Clinical features of patients in OME group and AH group

Thirty children (17 male and 13 female; mean age, 4.97 ± 1.19 years) were included in the OME group, and another thirty children (11 male and 19 female; mean age, 4.20 ± 1.27 years) were included in the AH group (Table 1). The degree of AH was III° in 7 cases and IV° in 23 cases in the OME group. In the AH group, 8 cases had III° hypertrophy and 22 cases had IV° hypertrophy. There were no statistically significant differences in age, gender, and adenoid size between the two groups (P > 0.05).

**TABLE 1:** Clinical features of patients.

Variable	OME group	AH group	P value
Age(years), mean (SD)	4.97 ± 1.19	4.20 ± 1.27	0.722
Sex			
Male	17	11	0.195
Female	13	19	
Degree of AH			
III°	7	8	1.000
IV°	23	22	

Data are presented as mean ± SD, or number of patients. P values were calculated using Student’s t-test for age and Chi-square test for sex and adenoid size. AH, adenoid hypertrophy; OME, otitis media with effusion; SD, standard deviation.

### 3.2 Comparison of IL-17 concentration in adenoid tissue and serum between OME group and AH group

**FIGURE 2:**
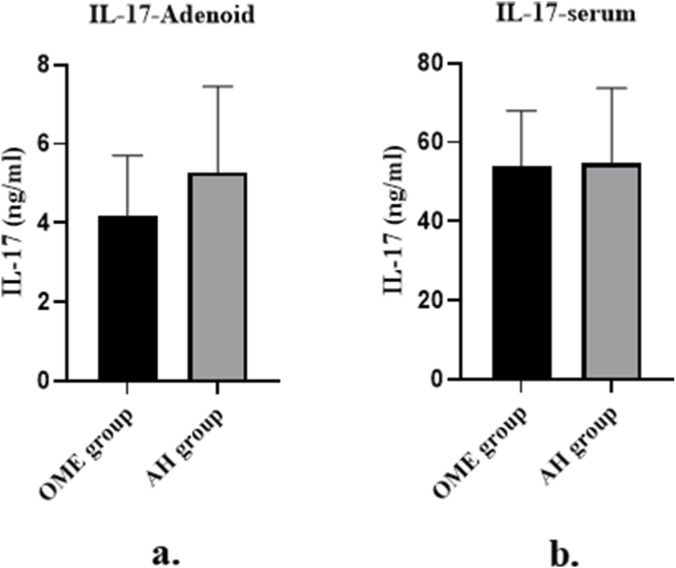
Comparison of IL-17 concentration in adenoid tissue and serum between OME group and AH group. **(a)** Bar chart showing IL-17 concentration in adenoid tissue of OME group (n = 30) and AH group (n = 30). Data are presented as mean ± SD. *P* = 0.037, Student’s t-test. **(b)** Bar chart showing IL-17 concentration in serum of OME group and AH group. Data are presented as mean ± SD. ns, not significant (P = 0.346, Student’s t-test).

The average IL-17 concentration in adenoid tissue of the OME group was 4.20 ± 1.51 ng/mL, which was significantly lower than that in the AH group (5.29 ± 2.16 ng/mL; P = 0.037, Figure 2a) (Table 2; Figure 2). No statistically significant difference was observed in serum IL-17 levels between the two groups (P = 0.346, Figure 2b).

**TABLE 2:** The results of IL-17 concentrations in the OME and AH groups.

Group	n	Adenoid tissue IL-17 (ng/mL)	Serum IL-17 (ng/mL)
OME group	30	4.20 ± 1.51	54.04 ± 13.87
AH group	30	5.29 ± 2.16	54.81 ± 18.84
Mean difference (95% CI)	​	1.09 (0.07–2.11)	0.77 (−7.84 to 9.38)
P value	​	0.037	0.859

Data are presented as mean ± SD. P values were calculated using Student’s t-test. CI, confidence interval; IL-17, interleukin-17; OME, otitis media with effusion; AH, adenoid hypertrophy.

### 3.3 Comparison of IL-10 concentration in adenoid tissue and serum between OME group and AH group

**FIGURE 3:**
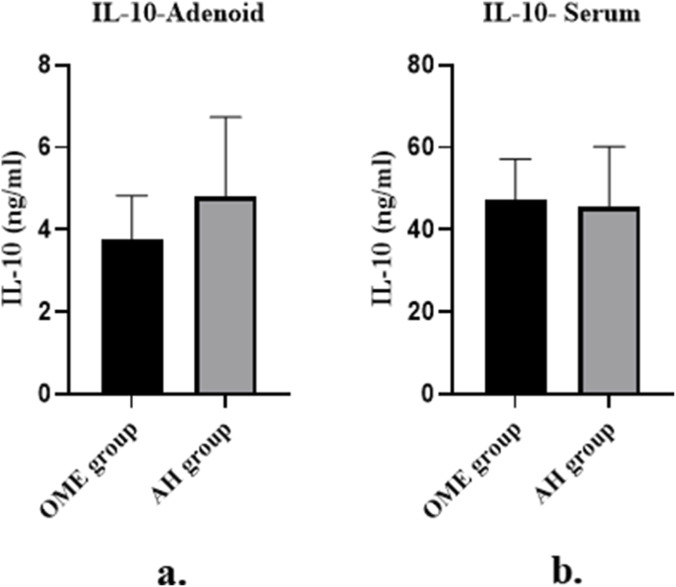
Comparison of IL-10 concentration in adenoid tissue and serum between OME group and AH group. **(a)** Bar chart showing IL-10 concentration in adenoid tissue of OME group (n = 30) and AH group (n = 30). Data are presented as mean ± SD. P = 0.009, Student’s t-test. **(b)** Bar chart showing IL-10 concentration in serum of OME group and AH group. Data are presented as mean ± SD. ns, not significant (P = 0.817, Student’s t-test).

The average IL-10 concentration in adenoid tissue of the OME group was 3.77 ± 1.06 ng/mL, which was significantly lower than that in the AH group (4.80 ± 1.93 ng/mL; P = 0.009, Figure 3a) (Table 3; Figure 3). No statistically significant difference was observed in serum IL-10 levels between the two groups (P = 0.817, Figure 3b).

**TABLE 3:** The results of IL-10 concentrations in the OME and AH groups.

Group	n	Adenoid tissue IL-10 (ng/mL)	Serum IL-10 (ng/mL)
OME group	30	3.77 ± 1.06	47.16 ± 9.99
AH group	30	4.80 ± 1.93	45.62 ± 14.77
Mean difference (95% CI)	​	1.03 (0.27–1.79)	−1.54 (−8.11 to –5.03)
P value	​	0.009	0.642

Data are presented as mean ± SD. P values were calculated using Student’s t-test. CI, confidence interval; IL-10, interleukin-10; OME, otitis media with effusion; AH, adenoid hypertrophy.

### 3.4 Predictive efficacy of adenoid tissue IL-17 and serum IL-17 for AH combined with OME

**FIGURE 4:**
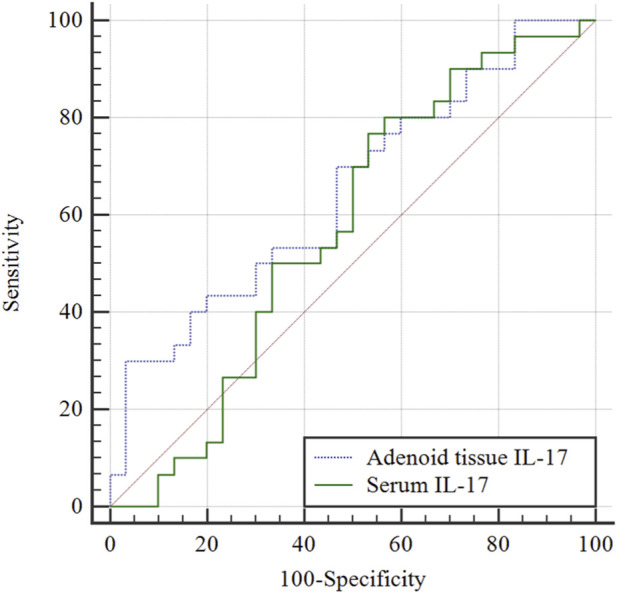
Predictive efficacy of adenoid tissue IL-17 and serum IL-17 for AH combined with OME. Through ROC curve analysis, the AUC and specificity of adenoid tissue IL-17 in predicting AH combined with OME were 0.649% and 96.67% respectively, which were superior to serum IL-17 (P < 0.05).

ROC curve analysis revealed that the AUC of adenoid tissue IL-17 in predicting AH combined with OME was 0.649 (95% confidence interval [CI]: 0.515–0.768, P = 0.037), with a specificity of 96.67% (95% CI: 82.8%–99.9%) and a sensitivity of 30.00% (95% CI: 15.4%–48.2%) (Table 4; Figure 4). The optimal critical value was ≤2.823 ng/mL, with a Youden index of 0.267. These values were superior to those of serum IL-17, which had an AUC of 0.572 (95% CI: 0.438–0.699, P = 0.346), with a sensitivity of 76.67% (95% CI: 57.7%–90.1%) and a specificity of 46.67% (95% CI: 28.3%–65.7%).

**TABLE 4:** Predictive efficacy of adenoid tissue IL-17 and serum IL-17 for AH combined with OME.

Indicator	AUC (95% CI)	Optimal critical value	Sensitivity % (95% CI)	Specificity % (95% CI)	*P* value	Youden index J
Adenoid tissue IL-17	0.649 (0.515–0.768)	≤2.823	30.00 (15.4–48.2)	96.67 (82.8–99.9)	0.037	0.267
Serum IL-17	0.572 (0.438–0.699)	≤63.89	76.67 (57.7–90.1)	46.67 (28.3–65.7)	0.346	0.233

AUC, area under the curve; CI, confidence interval; IL-17, interleukin-17; OME, otitis media with effusion; AH, adenoid hypertrophy.

### 3.5 Predictive efficacy of adenoid tissue IL-10 and serum IL-10 for AH combined with OME

**FIGURE 5:**
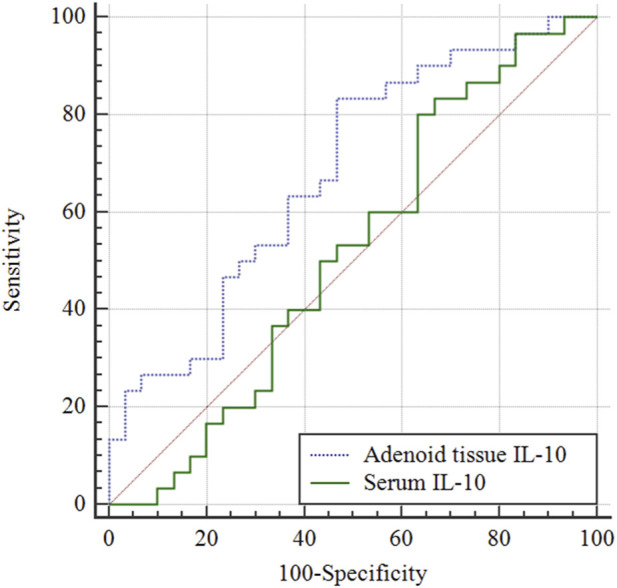
Predictive efficacy of adenoid tissue IL-10 and serum IL-10 for AH combined with OME. Through ROC curve analysis, the AUC, sensitivity and specificity of adenoid tissue IL-10 in predicting AH combined with OME were 0.681, 83.33% and 53.33% respectively, which were superior to serum IL-10 (P < 0.05).

ROC curve analysis revealed that the AUC of adenoid tissue IL-10 in predicting AH combined with OME was 0.681 (95% CI: 0.548–0.796, P = 0.009), with a sensitivity of 83.33% (95% CI: 65.3%–94.4%) and a specificity of 53.33% (95% CI: 34.3%–71.7%) (Table 5; Figure 5). The optimal critical value was ≤4.44 ng/mL, with a Youden index of 0.367. These values were superior to those of serum IL-10, which had an AUC of 0.518 (95% CI: 0.385–0.649, P = 0.817), with a sensitivity of 80.00% (95% CI: 61.4%–92.3%) and a specificity of 36.67% (95% CI: 19.9%–56.1%).

**TABLE 5:** Predictive efficacy of adenoid tissue IL-10 and serum IL-10 for AH combined with OME.

Indicator	AUC (95% CI)	Optimal critical value	Sensitivity % (95% CI)	Specificity % (95% CI)	P value	Youden index J
Adenoid tissue IL-10	0.681 (0.548–0.796)	≤4.44	83.33 (65.3–94.4)	53.33 (34.3–71.7)	0.009	0.367
Serum IL-10	0.518 (0.385–0.649)	≤54.47	80.00 (61.4–92.3)	36.67 (19.9–56.1)	0.817	0.167

AUC, area under the curve; CI, confidence interval; IL-10, interleukin-10; OME, otitis media with effusion; AH, adenoid hypertrophy.

### 3.6 Multivariate analysis of factors associated with OME in children with AH

To determine whether the association between lower adenoid tissue cytokine levels and OME was independent of potential confounders, we performed multivariate binary logistic regression analysis (Table 6). After adjusting for age, gender, and adenoid size, adenoid tissue IL-17 remained significantly associated with the presence of OME (adjusted OR = 0.712, 95% CI: 0.518–0.979, P = 0.038). Similarly, adenoid tissue IL-10 showed a significant independent association with OME after adjustment (adjusted OR = 0.634, 95% CI: 0.422–0.953, P = 0.028). Age, gender, and adenoid size were not significantly associated with OME in the multivariate model (all P > 0.05) (Table 6).

**TABLE 6:** Multivariate logistic regression analysis of factors associated with OME in children with AH.

Variable	Adjusted OR	95% CI	P value
Adenoid tissue IL-17	0.712	0.518–0.979	0.038
Adenoid tissue IL-10	0.634	0.422–0.953	0.028
Age	1.198	0.785–1.828	0.405
Gender (male vs. female)	0.658	0.214–2.023	0.467
Adenoid size (IV° vs. III°)	1.103	0.331–3.678	0.874

OR, odds ratio; CI, confidence interval. Adjusted ORs, represent the independent contribution of each variable after controlling for all other variables in the model. IL-17, interleukin-17; IL-10, interleukin-10; OME, otitis media with effusion; AH, adenoid hypertrophy.

## 4 Discussion

The adenoid is an important component of MALT, located behind the nasal cavity on the roof of the rhinopharynx, and plays a crucial role in developing immunity against inhaled microorganisms during early life. T cells in its lymphoid tissue can specifically recognize pathogens, initiate immune responses, and play a significant role in adenoid immune responses and pathogen clearance. Enlarged adenoids can cause narrowing of the upper airway, serving as a common etiology for sleep-disordered breathing (SDB) or obstructive sleep apnea-hypopnea syndrome (OSAS) in children. They can also act as a reservoir for pathogens, contributing to middle ear infections, recurrent rhinosinusitis, upper respiratory infections, and even invasive diseases such as pneumonia, bacteremia, and meningitis (Huang et al., 2019; Bogaert et al., 2004; H et al., 2024; Shin et al., 2008). The persistence of viral and bacterial pathogens may contribute to sustained inflammation in OME (Skovbjerg et al., 2020). Thus, the impact of adenoid immune function on childhood OME warrants attention. In 2011, Kotowski et al. (2011) indicated reduced T-cell activation in adenoids of patients with OME compared to those without OME. In 2012, Beata Zelazowska-Rutkowska’s study suggested a difference in the immunological responses between hypertrophic adenoids with otitis media and hypertrophic adenoids without otitis media (Zelazowska-Rutkowska et al., 2012b). In 2020, Zelazowska-Rutkowska et al. (2020) assessed the levels of VEGF-A and TGF-β cytokines in children with AH concomitant with OME and in children with AH alone. Currently, research on the relationship between adenoid immune status and childhood OME is limited, and no consensus has been reached.

*S. pneumoniae* is one of the major colonizers in the nasopharyngeal adenoids and the most common pathogen of otitis media in children (Emaneini et al., 2013). In 2013, Emaneini et al. (2013) found that *S. pneumoniae* colonizes the adenoid tissue, migrates to the middle ear cavity, and contributes to the overall pathogenesis of OME. Protection against pneumococcal colonization is mediated by IL-17A-producing Th17 cells (Malley, 2010). IL-17 recruits and activates neutrophils and macrophages to the nasopharynx, facilitating the clearance of pneumococci. In 2008, Lu et al. (2008) first proposed the association between Th17-mediated immune responses and the clearance of pneumococcal colonization in the nasopharynx. In 2015, Hoe et al. (2015) found that reduced IL-17A secretion was associated with high levels of pneumococcal nasopharyngeal carriage and concluded that IL-17 might be important in defending against pneumococcal colonization. In 2019, Huang et al. (2019) reported that in OME patients, prolonged or chronic pneumococcal carriage may occur due to insufficient IL-17A-mediated mucosal clearance, which could further be associated with OME development. In our study, the IL-17 concentration in adenoid tissue of the OME group was significantly lower than that of the AH group, while the IL-17 concentration in serum showed no significant difference between the two groups. This suggests that the low concentration of IL-17 in the local adenoid tissue, rather than in serum, leads to a decrease in the clearance ability of bacteria such as *S. pneumoniae* from the surface of adenoids. Ineffective or dysregulated IL-17 signaling may create a favorable microenvironment for *S. pneumoniae* to establish high-density colonization. Increased *S. pneumoniae* colonization causes damage to adjacent tissues, including the Eustachian tube and middle ear. Damage to the Eustachian tube can lead to blockage, which makes OME more likely to occur or persist. Additionally, *S. pneumoniae* could directly damage the middle ear mucosa, further promoting the occurrence and development of OME. Our study further elucidated the relationship between local IL-17 levels in adenoids and the pathogenesis of OME in children. Future novel therapeutics capable of regulating the local level of IL-17 in adenoid tissue may be beneficial for children with persistent or recurrent OME requiring repeated ventilation tube insertion.

As the key secretion of Treg cells, IL-10 is a critical anti-inflammatory cytokine that limits immune activation of innate and adaptive immune cells, which are related to the onset and development of chronic inflammatory diseases in the upper respiratory tract (Saraiva and O’Garra, 2010). In 2024, York et al. (2024) reported that IL-10 constrained sphingolipid metabolism to limit inflammation. As IL-10 downregulates inflammatory reactions, any imbalance in its production may induce a chronic inflammatory state (Zielnik-Jurkiewicz and Stankiewicz-Szymczak, 2016). Some studies have focused on the relationship between IL-10 and OME (Zielnik-Jurkiewicz and Stankiewicz-Szymczak, 2016; Skotnicka and Hassmann, 2000). Currently, studies on the relationship between IL-10 and AH concomitant with OME are few. In 2012, Zelazowska-Rutkowska et al. (2012a) found no differences in the secretion of IL-10 between the AH group and the OME group. In 2018, Feng et al. (2018) proposed that serum IL-21, IL-8, IL-6, IL-10, and IgE may be involved in the onset of OME in children. In contrast to these findings, our study showed that the concentration of IL-10 in the adenoid tissue of the OME group was significantly lower than that of the AH group, with a statistically significant difference. However, no significant difference in serum IL-10 concentration was found between the OME group and the AH group. This indicates that local low IL-10 concentration in adenoids may be associated with the development of OME in children. The possible mechanism is that a decrease in IL-10 in the local area of the adenoid reduces its anti-inflammatory effect, causing the adenoid to remain in a sustained inflammatory state, which makes the Eustachian tube and middle ear more susceptible to inflammatory stimulation. This may contribute to blockage of the Eustachian tube, directly or indirectly triggering an immune response in the middle ear and potentially being linked to the development of OME. Furthermore, IL-17 and IL-10 are important immune factors for Th17 and Treg cells, respectively, playing a crucial role in maintaining Th17/Treg immune balance. IL-10 is essential for maintaining immunological homeostasis (Abdel-Raoof Fouda et al., 2025). Low concentrations of IL-17 and IL-10 can disrupt the balance of Th17/Treg in the adenoids, reduce the immune protective effect of the adenoids on surrounding tissues, and make adjacent tissues such as the Eustachian tube/middle ear more susceptible to inflammation, which can further promote the occurrence and development of OME.

The robustness of our findings was further supported by multivariate logistic regression analysis (Table 6), which demonstrated that the associations between lower adenoid tissue IL-17 (adjusted OR = 0.712, 95% CI: 0.518–0.979, P = 0.038) and IL-10 (adjusted OR = 0.634, 95% CI: 0.422–0.953, P = 0.028) levels and the presence of OME were independent of age, gender, and adenoid size. This suggests that the local immune status of the adenoid tissue, rather than demographic factors or the degree of mechanical obstruction, plays a primary role in the pathogenesis of OME in children with AH. The adjusted odds ratios less than 1 indicate that higher cytokine levels are associated with lower odds of OME, consistent with our hypothesis that local immune deficiency contributes to disease development. Low levels of IL-17 and IL-10 may indicate a reduced ability of adenoids to clear bacteria, weakened immune suppression, and impaired immune regulation, which are associated with the occurrence and development of OME in children.

It is important to acknowledge the inherent limitation of the cross-sectional design in determining causal relationships. While our findings demonstrate a significant association between lower adenoid tissue IL-17 and IL-10 levels and the presence of OME, the direction of this association cannot be definitively established. An alternative explanation worthy of consideration is that the reduced cytokine levels may be a consequence, rather than a cause, of OME. Chronic middle ear inflammation and effusion could potentially induce systemic or local immunological changes that downregulate cytokine production in adjacent lymphoid tissues, including the adenoids. For instance, persistent inflammatory stimuli from the middle ear might lead to immune exhaustion or feedback inhibition of Th17 and Treg responses in the nasopharyngeal lymphoid tissue. Conversely, it is also plausible that a pre-existing local immune deficiency predisposes children to OME, and the subsequent inflammatory process further depletes or suppresses cytokine expression, creating a self-perpetuating cycle. Longitudinal studies with serial cytokine measurements before and after OME onset, or interventional studies in animal models, would be necessary to establish causality and elucidate the temporal sequence of these immunological events.

The limitation of this study is that the sample size is relatively small, and a larger sample size is necessary for more in-depth research. Through ROC curve analysis, the AUC and specificity of adenoid tissue IL-17 in predicting AH combined with OME were 0.649% and 96.67%, respectively, which were superior to serum IL-17 (P < 0.05). Through ROC curve analysis, the AUC, sensitivity, and specificity of adenoid tissue IL-10 in predicting AH combined with OME were 0.681, 83.33%, and 53.33%, respectively, which were superior to serum IL-10 (P < 0.05). This is mainly because the cytokines in tissue are more targeted and reflect the local pathophysiological state more accurately. The sources of IL-10 and IL-17 in serum are extensive (Yan et al., 2025). They not only come from adenoids and middle ear tissues, but also may come from immune cells and tissues in other parts of the body, and are affected by multiple factors during detection. It is worth noting, however, that the sensitivity of IL-17 detection in this organization is relatively low and may be affected by individual differences and cytokine diversity. Other indicators can be combined for collaborative observation during the detection.

## 5 Conclusion

The results of our study suggest that IL-17 and IL-10 in adenoid tissue may influence the immune function of hypertrophic adenoids. Low levels of IL-17 and IL-10 may indicate a reduced ability of adenoids to clear bacteria, weakened immune suppression, and impaired immune regulation, which are associated with the occurrence and development of OME in children. Regulating the local levels of IL-17 and IL-10 in adenoids may offer a novel approach for preventing and treating OME.

## Data Availability

The original contributions presented in the study are included in the article/supplementary material, further inquiries can be directed to the corresponding author.

## References

[B1] Abdel-Raoof Fouda M. Abdel-Wahhab M. Abdelkader A. E. Ibrahim M. E. Elsheikh T. A. Aldeweik H. M. (2025). Effect of gut microbiota changes on cytokines IL-10 and IL-17 levels in liver transplantation patients. BMC Infect. Dis. 25 (1), 140. 10.1186/s12879-025-10466-9 39885417 PMC11780876

[B2] Bogaert D. De Groot R. Hermans P. W. (2004). Streptococcus pneumoniae colonisation: the key to pneumococcal disease. Lancet Infect. Dis. 4 (3), 144–154. 10.1016/S1473-3099(04)00938-7 14998500

[B4] Emaneini M. Gharibpour F. Khoramrooz S. S. Mirsalehian A. Jabalameli F. Darban-Sarokhalil D. (2013). Genetic similarity between adenoid tissue and middle ear fluid isolates of Streptococcus pneumoniae, Haemophilus influenzae and Moraxella catarrhalis from Iranian children with otitis media with effusion. Int. J. Pediatr. Otorhinolaryngol. 77 (11), 1841–1845. 10.1016/j.ijporl.2013.08.024 24080321

[B5] Feng C. Zhang Q. Zhou G. Zhang J. Zhang Y. (2018). Roles of T follicular helper cells in the pathogenesis of adenoidal hypertrophy combined with secretory otitis media. Med. (Baltimore) 97 (13), e0211. 10.1097/MD.0000000000010211 29595664 PMC5895434

[B6] Hu L. He W. Li J. Miao Y. Liang H. Li Y. (2024). The role of adenoid immune phenotype in polysensitized children with allergic rhinitis and adenoid hypertrophy. Pediatr. Allergy Immunol. 35 (6), e14166. 10.1111/pai.14166 38822736

[B7] Hoe E. Boelsen L. K. Toh Z. Q. Sun G. W. Koo G. C. Balloch A. (2015). Reduced IL-17A secretion is associated with high levels of pneumococcal nasopharyngeal carriage in Fijian children. PLoS One 10 (6), e0129199. 10.1371/journal.pone.0129199 26069966 PMC4466549

[B8] Huang C. C. Wu P. W. Lee T. J. Chen C. L. Wang C. H. Tsai C. N. (2019). Differential IL-17A response to S. pneumoniae in adenoid tissue of children with sleep disordered breathing and otitis media with effusion. Sci. Rep. 9 (1), 19839. 10.1038/s41598-019-56415-w 31882693 PMC6934741

[B9] Jochems S. P. Weiser J. N. Malley R. Ferreira D. M. (2017). The immunological mechanisms that control pneumococcal carriage. PLoS Pathog. 13 (12), e1006665. 10.1371/journal.ppat.1006665 29267378 PMC5739485

[B10] Kotowski M. Niedzielski A. Niedzielska G. Lachowska-Kotowska P. (2011). Dendritic cells and lymphocyte subpopulations of the adenoid in the pathogenesis of otitis media with effusion. Int. J. Pediatr. Otorhinolaryngol. 75 (2), 265–269. 10.1016/j.ijporl.2010.11.014 21144597

[B12] Lu Y. J. Gross J. Bogaert D. Finn A. Bagrade L. Zhang Q. (2008). Interleukin-17A mediates acquired immunity to pneumococcal colonization. PLoS Pathog. 4 (9), e1000159. 10.1371/journal.ppat.1000159 18802458 PMC2528945

[B13] Malley R. (2010). Antibody and cell-mediated immunity to Streptococcus pneumoniae: implications for vaccine development. J. Mol. Med. 88, 135–142. 10.1007/s00109-009-0579-4 20049411

[B14] Ni K. Zhao L. Wu J. Chen W. Yang H. Li X. (2015). Th17/Treg balance in children with obstructive sleep apnea syndrome and the relationship with allergic rhinitis. Int. J. Pediatr. Otorhinolaryngol. 79 (9), 1448–1454. 10.1016/j.ijporl.2015.06.026 26166452

[B15] Qureishi A. Lee Y. Belfield K. Birchall J. P. Daniel M. (2014). Update on otitis media—prevention and treatment. Infect. Drug Resist. 7, 15–24. 10.2147/IDR.S39637 24453496 PMC3894142

[B16] Sade K. Fishman G. Kivity S. DeRowe A. Langier S. (2011). Expression of Th17 and Treg lymphocyte subsets in hypertrophied adenoids of children and its clinical significance. Immunol. Invest. 40 (6), 657–666. 10.3109/08820139.2011.575426 21542720

[B17] Saraiva M. O’Garra A. (2010). The regulation of IL-10 production by immune cells. Nat. Rev. Immunol. 10 (3), 170–181. 10.1038/nri2711 20154735

[B18] Shin K. S. Cho S. H. Kim K. R. Tae K. Lee S. H. Park C. W. (2008). The role of adenoids in pediatric rhinosinusitis. Int. J. Pediatr. Otorhinolaryngol. 72 (11), 1643–1650. 10.1016/j.ijporl.2008.07.016 18789545

[B19] Siegel S. J. Weiser J. N. (2015). Mechanisms of bacterial colonization of the respiratory tract. Annu. Rev. Microbiol. 69, 425–444. 10.1146/annurev-micro-091014-104209 26488280 PMC4760621

[B20] Skotnicka B. Hassmann E. (2000). Cytokines in children with otitis media with effusion. Eur. Arch. Otorhinolaryngol. 257 (6), 323–326. 10.1007/s004059900218 10993552

[B21] Skovbjerg S. Roos K. Andersson M. Rabe H. Nilsson S. Lindh M. (2020). Inflammatory mediator profiles in secretory otitis media in relationship to viable bacterial pathogens and bacterial and viral nucleic acids. J. Interferon Cytokine Res. 40 (12), 555–569. 10.1089/jir.2020.0075 33337936

[B23] Yan Y. Wang Y. Song Y. Cui L. Wang L. Liu X. (2025). Role of laryngopharyngeal reflux in the etiology of otitis media with effusion in children. Eur. Arch. Otorhinolaryngol. 282, 1–6. 10.1007/s00405-025-09402-z 40281316

[B24] York A. G. Skadow M. H. Oh J. Qu R. Zhou Q. D. Hsieh W. Y. (2024). IL-10 constrains sphingolipid metabolism to limit inflammation. Nature 627 (8004), 628–635. 10.1038/s41586-024-07098-5 38383790 PMC10954550

[B25] Zelazowska-Rutkowska B. Skotnicka B. Cylwik B. (2020). Vascular endothelial growth factor and transforming growth factor β in hypertrophic adenoids in children suffering from otitis media with effusion. Cytokine 133, 155125. 10.1016/j.cyto.2020.155125 32438279

[B26] Żelazowska-Rutkowska B. Wysocka J. Ratomski K. Kasprzycka E. Skotnicka B. (2012a). Increased percentage of T cells with the expression of CD127 and CD132 in hypertrophic adenoid in children with otitis media with effusion. Eur. Arch. Otorhinolaryngol. 269 (7), 1821–1825. 10.1007/s00405-012-1977-8 22382400 PMC3365238

[B27] Zelazowska-Rutkowska B. Ilendo E. Skotnicka B. Wysocka J. Kasprzycka E. (2012b). Production of cytokines by mononuclear cells of hypertrophic adenoids in children with otitis media with effusion. Folia Histochem Cytobiol. 50 (4), 586–589. 10.5603/20326 23264223

[B28] Zhang Q. Leong S. C. McNamara P. S. Mubarak A. Malley R. Finn A. (2011). Characterisation of regulatory T cells in nasal associated lymphoid tissue in children: relationships with pneumococcal colonization. PLoS Pathog. 7 (8), e1002175. 10.1371/journal.ppat.1002175 21852948 PMC3154846

[B30] Zielnik-Jurkiewicz B. Stankiewicz-Szymczak W. (2016). Evaluation of the Interleukin-1 receptor antagonist and immunoregulatory Interleukin-10 in the middle ear in chronic otitis media with effusion in children with and without atopy. Clin. Exp. Otorhinolaryngol. 9 (2), 104–108. 10.21053/ceo.2015.00129 27090281 PMC4881322

